# On Painful Menstruation

**Published:** 1891-09

**Authors:** 


*On Painful Menstruation.* The Harveian Lectures, 1890. By FRANCIS HENRY CHAMPNEYS, M.A., M.D. Oxon., F.R.C.P. Pp. viii.—88. London: H. K. LEWIS. 1891.

By the aid of large type of an excellent fount, and of thick paper which it is a pleasure to handle, these lectures form an imposing and handsome volume. The subject chosen by Dr. Champneys is of great practical interest and, as befitted the occasion upon which the lectures were delivered, is dealt with scientifically and with dignity; its literature, so copious that a special receptacle had to be provided for the accommodation of the large tables in which a summary of it is presented, is considered in detail and in an interesting manner. Dealing largely in theory, the author's statements are often open to criticism, but the arguments of other observers are fairly given and dispassionately examined.

Importance is rightly attached to the need of a careful differential diagnosis in all cases of so-called membranous dysmennorrhœa especially in reference to the products of conception; and, as its treatment is said to be "a most unhappy problem," diligent search must be made for the cause, which the author thinks will usually be found to be of an inflammatory character. Dr. Champneys, firmly convinced that spasmodic dysmennorrhœa is a neurosis, enters a vigorous protest against treatment much too frequently based on a mechanical system of pathology, although, in the absence of the great remedy of pregnancy and parturition, he would, as a last resort, have recourse to dilatation, which, if adopted, he recommends to be carried out at one sitting under anæsthesia, and with full antiseptic precautions.

Dr. Champneys' language is occasionally epigrammatic; and among his best sayings is the one that "in matters of science Englishmen live less on an island than other people;" but when he prints the name of the dedicatee of his book in a Latinised form he gives it an appearance which is decidedly comic.

A full table of contents to the volume is supplied, but no index.

